# Influence of Effective Water-to-Cement Ratios on Internal Damage and Salt Scaling of Concrete with Superabsorbent Polymer

**DOI:** 10.3390/ma12233863

**Published:** 2019-11-22

**Authors:** Minsoo Kim, Sung-Hoon Kang, Sung-Gul Hong, Juhyuk Moon

**Affiliations:** 1 Department of Architecture & Architectural Engineering, Seoul National University, 1 Gwanak-ro, Gwanak-gu, Seoul 08826, Koreamedesis@snu.ac.kr (S.-H.K.); 2 Department of Civil & Environmental Engineering, Seoul National University, 1 Gwanak-ro, Gwanak-gu, Seoul 08826, Korea

**Keywords:** superabsorbent polymer, absorbency, capillary absorption, freezing and thawing

## Abstract

Superabsorbent polymer (SAP) is attracting attention as a water-entraining admixture that reduces shrinkage or heals cracks in concrete. Cross-linked sodium polyacrylate SAPs, which are the most widely produced SAPs in the global market, are applicable as concrete admixtures. However, there have been contradictory results on the freeze–thaw resistance of concrete with SAPs. This study aims to clarify these results considering the water absorption behavior of SAPs in hardened concrete when effective water-to-cement ratios are different. Firstly, the absorbencies of one kind of cross-linked sodium polyacrylate SAP (SAP_SP) in pore solution and fresh mortar were measured by a tea bag test and flow test, respectively. Pore size distribution, capillary water absorption, and deformation during freeze–thaw cycles were analyzed for mortar samples with varying SAP_SP dosages. In the main tests, concrete samples with three different SAP_SPs/cement ratios (0.1%, 0.2%, and 0.3%) and a reference sample were prepared, and internal damage and salt scaling were measured under freeze–thaw cycles. Because SAP_SP absorbs water in fresh mixtures, additional water was added to the mixture considering the water absorbency of the SAP_SP. It was found that the used SAP_SPs prematurely release their stored water so the effective water-to-cement ratio was increased when a larger amount of SAP_SP was used. The higher effective water-to-cement ratio caused more internal damage and salt scaling due to the weaker cementitious matrix. In addition, mortar samples with a high SAP_SP content show a larger absorption of capillary water than the reference sample. The result can be interpreted by an observation that SAP_SP in air voids absorbs water and expands to relatively large capillary pores or neighbor air voids during the capillary water absorption process.

## 1. Introduction

Superabsorbent polymers (SAPs) are powdery products that absorb water of masses several tens or hundreds of times their own mass. Studies have reported on SAP-induced changes in cement composite properties after Jensen et al. proposed SAP as a water-entraining admixture for internal curing in high-performance concrete [1,2]. It has been reported that SAPs can reduce the permeability of water through cracks when cracks occur in cement composites [3,4,5,6]. It also has been reported that SAPs heal cracks by supplying moisture to cement composites [7,8,9,10].

Studies on the effect of SAPs on the freeze–thaw resistance of concrete have also been reported. SAPs that absorb water in fresh concrete release the water when the humidity inside the concrete decreases due to the hydration of concrete and the evaporation of water. The water release reduces the volume of the SAP and creates an air void inside the concrete. This air void is expected to improve the freeze–thaw resistance of concrete. Similarly, the pore structure of concrete mixed with various types of SAPs and the freeze–thaw resistance of concrete containing SAPs have been studied [11]. Freeze–thaw resistance tests for concrete have been conducted by setting the SAP dosage and SAP particle size as variables [12]. Recently, the effect of two types of SAPs on the freeze–thaw resistance of concrete in various regions of the world has been reported via the results of an inter-laboratory study [13]. In those studies, reference mixtures with different water-to-cement ratios were compared with respect to the freeze–thaw resistance. However, the effect of mixtures incorporating SAPs with different effective water-to-cement ratios (i.e., total-water-to-cement ratio minus water-stored-by-SAP-to-cement ratio) has not been fully elucidated. In this study, mixtures incorporating SAP with different effective water-to-cement ratios and SAP content have been studied with respect to pore size distribution, capillary water absorption, expansion of mixtures under freezing, and freeze–thaw resistance.

Along with the issue of effective water-to-cement ratios, there have been contradictory results regarding the freeze–thaw resistance of concrete with SAPs. Many experimental studies have found that SAP improves the freeze–thaw resistance of concrete [11,12,13,14,15]; however, opposite cases have also been reported [13,14,16]. A possible explanation for these conflicting results could be the complex absorption behavior of SAPs in hardened concrete when the concrete is exposed to water. This absorption and further desorption behavior will change the effective water-to-cement ratio, which is treated as the most important parameter in the mix design of concrete.

When hardened concrete is exposed to water, capillary pores (pores with a diameter of less than 10 μm) are known to be filled with water via capillary pressure. However, air in the air voids (pores with a diameter larger than 10 μm) is trapped during capillary absorption [17]. In the case of air in the air voids formed by SAPs, it will be trapped as well. The water in the capillary pores around an air void will continuously evaporate until the water in the capillary pores and the water vapor in the air void reach a thermodynamic equilibrium. A dry SAP in the air void will be exposed to this environment. Furthermore, the absorption behavior of SAPs in hardened concrete may vary depending on the type and size of the SAPs.

Common types of SAPs include cross-linked acylamide/acrylic acid copolymers, cross-linked sodium polyacrylate, polymethacrylic acid, and polyethylene oxide. Among these, cross-linked acylamide/acrylic acid copolymer is easy to manufacture and has a relatively high absorption rate in the cement pore solution environment whereas cross-linked sodium polyacrylate is mostly used for personal hygiene products. In our study, a cross-linked sodium polyacrylate (SAP_SP) which is generally the most inexpensive was selected to clarify its effect on concrete materials under freeze–thaw cycles. Because SAP_SP generally absorbs water in fresh mixtures, additional water should be added if considering the SAP’s absorbency. However, it was found that the used SAP_SP prematurely released the stored water. Thus, the effective water-to-cement ratios were increased with higher SAP_SP contents. Therefore, it has to be pointed out that experimental results obtained from the used SAP_SP may not be directly comparable to the available experimental data based on other types of SAPs with different absorption and desorption characteristics.

As an outline of the test program, the SAP_SP absorbencies in pore solution and fresh mortar were measured through a tea bag test and mortar flow test, respectively. The amount of capillary water absorption and pore size distribution were measured in mortar samples with effective water-to-cement ratio and SAP_SP dosage as variables. Moreover, the deformation of mortar samples during freeze–thaw cycles was recorded. As a main discussion point, the measured internal damage and salt scaling of concrete samples are considered.

## 2. Experimental Procedure

### 2.1. Preparation of Mortar and Concrete Samples

The samples were made from type I ordinary Portland cement (OPC-type I, Hanil cement, Seoul, Korea). The chemical oxide composition of the used cement can be found in our previous study [18]. Silica sand with a density of 2.67 g/mL and a fineness modulus of 2.46 was used as a fine aggregate. Gravel with a density of 2.94 g/mL and a diameter of 5–19 mm was used as a coarse aggregate. The samples were prepared at three SAP/cement ratios (0.1, 0.2, and 0.3 mass %); a reference sample without SAP was also prepared. Table 1 shows the mix proportions of the samples. Values of w/c_(add)_ were estimated considering the SAP_SP absorbency, which was measured during the tea bag test. However, SAP_SP prematurely releases stored water. As a result, the SAP_SP absorbency obtained from the flow test was observed to be much lower than the value from the tea bag test. The flow test may be more reasonably used for discussion considering the actual impact of the SAP_SP absorbency in mixtures. Thus, values of w/c_(stored by SAPs)_ were determined considering SAP_SP absorbency on the basis of the flow test. Values of w/c_(effective)_ were determined to be w/c_(total)_ minus w/c_(stored by SAPs)_. As a result, the calculated w/c_(effective)_ values were different for all samples as the higher the SAP_SP content the the higher w/c_(effective)_. This was set as the main experimental variable in this study.

In preparing the mortar samples, a mortar mixer with a paddle that revolves in a planetary motion was used. Cement was mixed with silica sand for 5 min and then mixed with dry SAP_SP powders for 2 min. Subsequently, water and polycarboxylate ether (PCE)-type superplasticizer (FLOWMIX P-STD, Dongnam, Pyeongtaek, Korea) were added into the mixtures and then mixed for 5 min. In preparing the concrete samples, mortar and gravels were weighed and then mixed for 10 min. The samples were sealed with a polyester film after casting, demolded after 24 h, and stored in a climate chamber (20 °C and relative humidity (RH) 60%) until tests were performed. The American Society for Testing and Materials (ASTM) C231 test was performed to measure the air content of the fresh concrete. The test results are shown in Table 2.

### 2.2. Characterization of SAPs 

**Figure 1:**
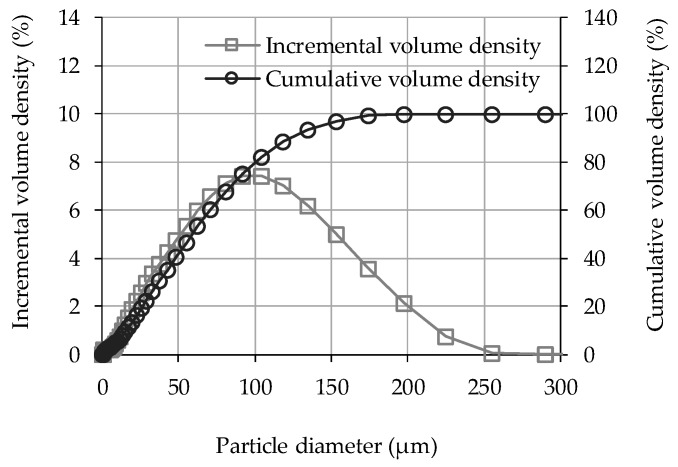
Incremental and cumulative particle size distributions of SAP_SP.

Commercially available SAPs (GE 500F-2, LG Chem, Seoul, Korea) were used. The main composition (92–100%) of the product was cross-linked sodium polyacrylate (–CH_2_–CH(CO_2_Na)), which was prepared through bulk solution polymerization. Irregular particles had a density of 1.5 g/mL. Size distribution of the particles was measured by laser diffraction technique (Mastersizer 3000, Malvern Panalytical, Malvern, UK) using ethanol as a dispersant, and the results are shown in Figure 1 [18,19]. The volume-based mean diameter D[4,3] was 69 μm.

The absorption characteristics of the SAP_SPs were measured using two methods. The first method used was a modified tea bag test [18]. In this method, a tea bag containing a specified mass of the SAP_SPs was immersed in a test solution and then the change in the tea bag mass was measured. In preparing the test solution, cement paste with a water/cement ratio of 0.45 was mixed. The fresh cement paste was poured into a stainless-steel filter holder (XX4004740, Merck, Burlington, MA, USA) and then nitrogen gas was applied at a pressure of 0.2 MPa to extract the solution through the filter (JHWP04700, Merck, Burlington, MA, USA). The solution was refrigerated (0–5 °C) until the tea bag test was performed. In the test, the mass of an empty tea bag (*M*_1_) was measured and 0.02 g (*M*_0_) of dry SAP_SPs was placed into the tea bag, which was then submerged in the prepared solution. Given the limited amount of the prepared solution, a small amount of the SAP_SPs was used. After a certain period of time, the tea bag was removed from the solution, wiped with a dry cloth, and centrifuged at 1000 rpm for 1 min; finally, the mass of the tea bag (*M*_2_) was measured. The solution absorbency of the SAP_SPs was calculated as follows: (*M*_2_ − *M*_1_ − *M*_0_)/*M*_0_. The absorbencies after 1, 10, 30, 60, and 180 min were obtained; one tea bag was prepared for each measurement.

The other method used was the mortar flow test, which was employed to observe the rheological changes due to SAP incorporation [20]. SAPs in fresh mortar absorb water to decrease the slump flow. Assuming the water absorbed by the SAP does not contribute to slump flow, the amount of absorbed water can be determined by the difference in total water between the reference mortar and the SAP-incorporated mortar when both are the same in slump flow. Then, the solution absorbency of the SAP_SPs can be estimated as (*M_w_*_2_ − *M_w_*_1_)/*M_SAP_*, where *M_w_*_2_ is the mass of the water added into the mortar containing the SAP_SPs, *M_w_*_1_ is the mass of the water added into the reference mortar, *M_SAP_* is the mass of the dry SAP_SPs added into the mortar containing SAP_SPs. In the test, the slump flow of mortar was measured at 5, 10, and 20 min after the first contact with water, and the mortar was mixed again for the first 1 min of the intervals.

### 2.3. Experimental Methods

**Figure 2:**
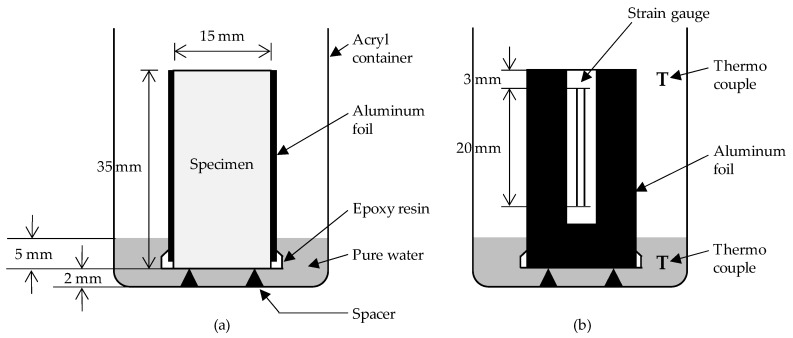
(**a**) Capillary suction test setup for mortar (section). (**b**) Strain measurement test setup for mortar during freeze–thaw cycles (elevation).

Mortar samples cured for 28 days were prepared for mercury intrusion porosimetry (MIP). Although the water saturation method (WSM) is applicable to determine open pore volumes [21], MIP was performed to determine pore size distribution. Mortar samples cured for 28 days with dimensions of 15 mm × 15 mm × 35 mm were prepared for a capillary suction test (Figure 2a). The samples were dried at 60 °C for 1 week until the test. Three replicates were tested for one measurement. The acryl container was placed in a leak-proof container to reduce water evaporation. The water depth was maintained at 7 mm through constant addition of water. The mass difference of the mortar samples was measured for 7 days from the time of water exposure. After a few preparations, the samples were also used to measure the strain of the mortar during freeze–thaw cycles (Figure 2b). A part of aluminum foil covering the surface of the samples was removed for a strain gauge attachment. Then, the sample was placed back into the water. Six h after the preparations, the acryl container was placed in a leak-proof container to reduce the evaporation of water and then placed in an environmental chamber, and four freeze–thaw cycles were performed. The temperature curve of the air in the acryl container was consistent with CEN/TS 12390-9 [22]. Importantly, the cooling rate from 0 °C to −20 °C was set to about −2.5 °C/h.

Concrete samples used in the freeze–thaw resistance test were prepared according to CEN/TS 12390-9, but the process that involves curing the samples in tap water was excluded to avoid the SAP_SPs from absorbing tap water at the early curing stage. The samples (150 mm × 150 mm × 50 mm) were obtained through wet sawing from 150 mm cubes on the 21st day and then stored in a climate chamber (20 °C and RH 60%). Four samples were prepared for each series. Before the 28th day, rubber cloths were glued onto all surfaces except one surface (the test surface) of the samples and a silicon string was placed on the edge of the test surface. Pure water was poured onto the test surface to a depth of 3 mm at 28 days from the start of the experiment. This condition lasted for 3 days at 20 °C. At 31 days, 3 mass % NaCl solution was poured onto the test surface to a depth of 3 mm before the relative dynamic modulus of the samples was measured. Except for the test surface, all surfaces of the samples were insulated with 20 mm thick polystyrene cellular plastic. The samples were placed in an environmental chamber and then subjected to freeze–thaw cycles. After 7, 14, 28, 42, and 56 cycles, the mass of fragments scaled from the test surface and the relative dynamic modulus of the samples were measured.

## 3. Results

### 3.1. Absorption Rate of SAPs in Concrete

**Figure 3:**
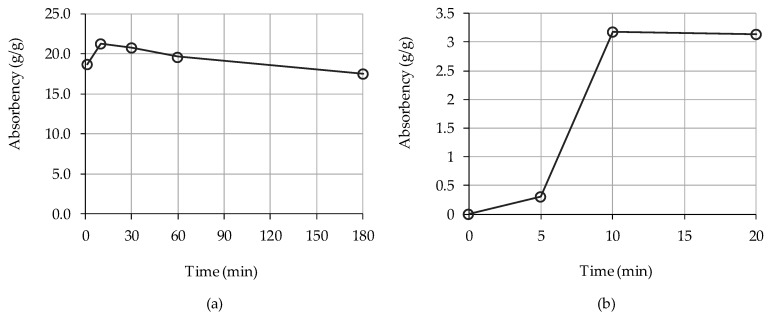
Absorbencies of the SAP_SPs in solution according to absorption time as measured by (**a**) tea bag test and (**b**) flow test.

Figure 3a shows the absorbency of the SAP_SPs from the tea bag test. The highest absorbency was 21.3 g/g at 10 min, which gradually decreased with time. However, it was found that SAP_SP prematurely released the stored water. The absorbency from the flow test was much lower than that obtained from the tea bag test. This can be explained by the actual impact of the absorbency of SAP_SP in mortar samples on the rheological changes which can be different from the absorbance characteristics of SAP_SP particles themselves. The highest absorbency of the SAP_SPs in fresh mortar was 3.1 g/g, which was observed at 10 min (Figure 3b).

### 3.2. Pore Size Distribution

**Figure 4:**
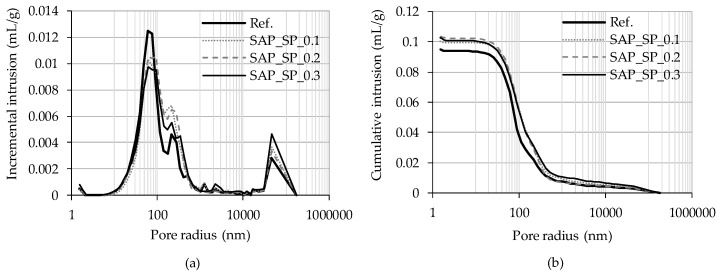
Pore size distribution of the mortar samples. (**a**) Incremental intrusion and (**b**) cumulative intrusion according to pore radius.

The bulk and skeletal densities and porosity of mortar samples as measured by an MIP test are shown in Table 3. The bulk density of the mortars containing the SAP_SPs were slightly smaller than that of the reference mortar (Ref.). The capillary pore structure of mortars containing large amounts of SAPs (for which the range of w/c_(stored by SAPs)_ was 0.04–0.3) has been studied by Snoeck et al. [23,24]. Their research showed that the capillary porosity of a reference mortar and mortars with SAPs are nearly the same, with the same w/c_(effective)_ being observed, although the water stored by SAPs was observed to cause further hydration of the cement, resulting in a small reduction in porosity. In the MIP test of the present study, porosity tended to increase with increasing w/c_(effective)_. Given the considerably low w/c_(stored by SAPs)_ values observed in this study, the hydration caused by the SAP_SPs was found to be negligible. The skeletal density showed no meaningful difference between samples. Figure 4 shows the pore size distribution of the mortar samples. The pore volume per unit mass of the Ref. sample is larger than that of the samples containing the SAP_SPs at a pore radius of 40–90 nm. On the contrary, when the pore radius is 90–400 nm or larger than 30 μm, the pore volume per unit mass of the samples containing the SAP_SPs can be seen to be larger than that of the Ref.

### 3.3. Capillary Water Absorption

**Figure 5:**
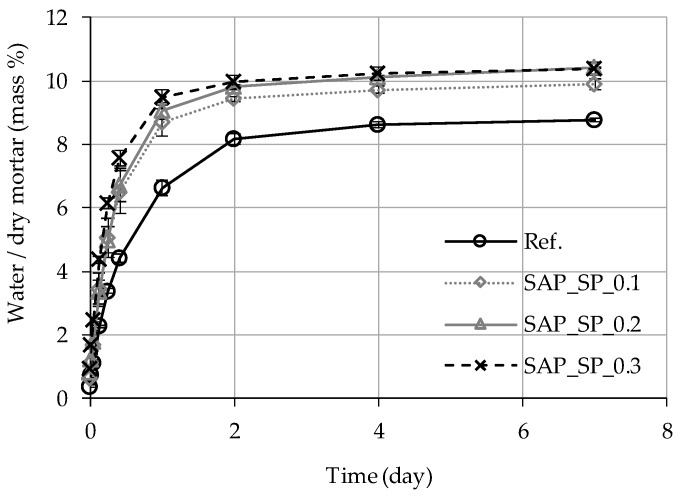
Capillary water absorption of mortar samples according to time immersed in water.

When a dry mortar sample was placed in water at a 5 mm depth, the sample absorbed water via capillary pressure. As shown in Figure 5, the higher the SAP_SP content (i.e., the higher the w/c_(effective)_), the faster the water absorption; this trend can be observed up to 6 h since the sample was first exposed to water. Between the first and the second day, the Ref. sample can be seen to absorb water more rapidly than the other samples. From the second day, the water absorption rate of all the samples slowed down. On the seventh day, the higher the SAP_SP content (i.e., the higher the w/c_(effective)_,), the larger the total mass of water absorbed.

### 3.4. Strain on Mortar During Freeze–Thaw Cycles

**Figure 6:**
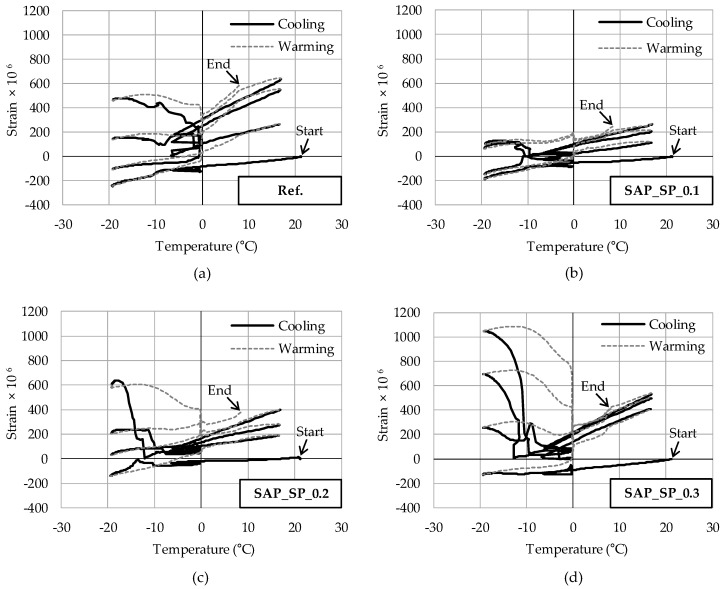
Strain on mortar samples (**a**) Ref. (**b**) SAP_SP_0.1 (**c**) SAP_SP_0.2 (**d**) SAP_SP_0.3 during the four freeze–thaw cycles.

The strain of three samples was measured for each series during the freeze–thaw cycles. Figure 6 shows the sample with the largest strain for each series as a function of water temperature in the acryl container. In the first cycle, all strains can be seen to decrease with a similar slope in the freezing phase. In the second cycle, differences in strain can be observed depending on the sample. The strain of SAP_SP_0.3 increases in the freezing phase, whereas that of the other samples decreases. In the third cycle, the strain of SAP_SP_0.3 increases significantly in the freezing phase, whereas the strains of SAP_SP_0.2 and Ref. increase only slightly. Finally, the peak points of the strain for SAP_SP_0.3, SAP_SP_0.2, Ref, SAP_SP_0.14, and SAP_SP_0.1 in the freezing phase of the fourth cycle are 1047 × 10^−6^, 637 × 10^−6^, 479 × 10^−6^, 369 × 10^−6^, and 129 × 10^−6^, respectively. Table 4 shows the average peak points of the strains of the three samples for each series during the freezing phase of the fourth cycle. The results show that SAP_SP_0.3 and SAP_SP_0.2, which have higher w/c_(effective)_ than the other samples, expand more as the freeze–thaw cycle repeats.

### 3.5. Freeze–Thaw Resistance of Concrete

**Figure 7:**
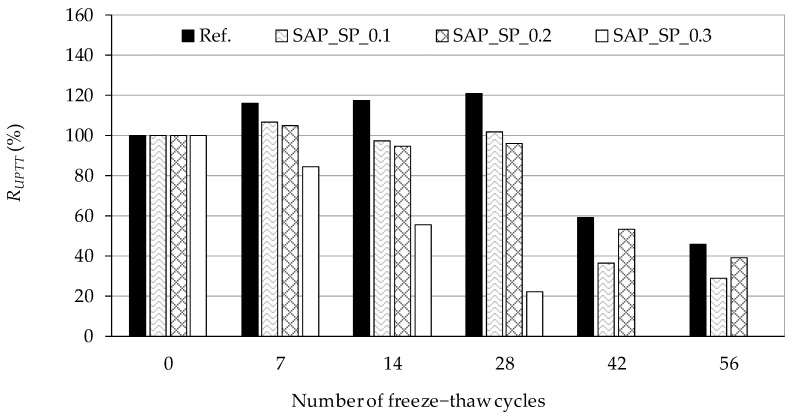
Relative dynamic modulus (*R_UPTT_*) of samples according to the number of freeze−thaw cycles.

The relative dynamic modulus of the concrete samples according to the number of freeze–thaw cycles is shown in Figure 7. In CEN/TS 12390-9, the sample is defined as damaged when the relative dynamic modulus of the sample is less than 80%. Ref. can be seen to always show the largest relative dynamic modulus, but after 56 cycles, the value becomes 46%, indicating that the sample experiences critical damage. In the early cycles there was a temporal increase of the dynamic modulus of a few samples. Stiffness enhancement of concrete by absorbing freezing water may cause the increase prior to the intended freezing-induced damage on samples. For SAP_SP_0.3, which has the highest w/c_(effective)_, the relative dynamic modulus after 28 cycles is the smallest, at 22%, and no stable signal was obtained after 42 and 56 cycles.

Although SAP_SP_0.2 shows a larger relative dynamic modulus than SAP_SP_0.1 after 42 and 56 cycles, the overall results show that the higher the SAP_SP content, the higher the w/c_(effective)_, and the faster the relative dynamic modulus decreases as the cycle repeats. 

**Figure 8:**
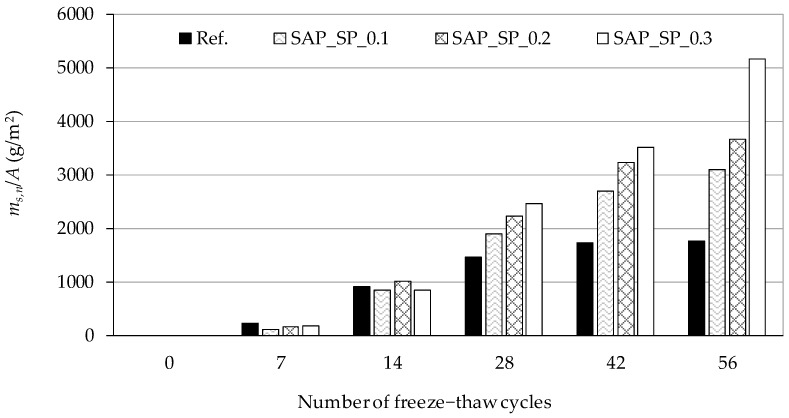
Cumulative mass of fragments scaled from unit area of sample surface (*m_s,n_/A*) according to the number of freeze–thaw cycles.

The cumulative mass of fragments scaled from the surface of the samples according to the number of freeze–thaw cycles is shown in Figure 8. The *m_s,n_*/*A* value was calculated according to CEN/TS 12390-9, wherein the cumulative mass of fragments was scaled from a unit area of the test surface. The results show that the higher the w/c_(effective)_, the larger the mass of fragments scaled from the sample. In SAP_SP_0.3, the mass of fragments scaled from the sample after 56 cycles can be seen to have significantly increased compared to the other samples.

## 4. Discussion

### 4.1. Air Voids Formed by SAPs

To discuss the characteristics of air voids formed by the SAP_SPs, the quantitative properties of the air voids in a unit volume of mortar were calculated. First, to estimate the mass of dry SAP_SPs in a unit volume of the mortar as shown in Table 5, the mass ratio of dry SAP_SPs in the fresh mortar in Table 1 was divided by the density of hardened mortar in Table 3. In this calculation, the bulk density of the hardened mortar was assumed to be equal to the density of fresh mortar. The volume of air voids formed by the SAP_SPs in a unit volume of mortar was estimated as in Table 5 by the volume of water stored by the SAP_SPs (absorbency = 3.1 g/g) in fresh mortar from the flow test.

### 4.2. Water Absorption in Capillary Pores and Air Voids

Once a dry mortar sample was placed in water at a 5 mm depth, the sample began to absorb water through its capillary pores (pores with a diameter of less than nm) due to capillary pressure. Fagerlund [17] explains the mechanism of capillary water absorption in mortar. In the absorption process, relatively small capillary pores are filled with water quickly, whereas air is trapped in relatively large capillary pores or air voids. The air is compressed by the surface tension of the water surrounding the air. The smaller the radius of the air bubble in the pore as determined based on the pore radius, the greater the compression and the smaller the air volume. For example, if the pore radius is 1000 nm, the trapped air volume will be 32% of the original volume, 68% of the original volume will be occupied by water, and the pressure in the air bubble will increase to 3.1 times the initial pressure. Compressed air can dissolve into pore water through the increased solubility of the air dissolved into the surrounding water, and this happens when the pressure of the air increases. Therefore, assuming that the dissolved air in the water does not escape out of the sample, capillary water absorption can be predicted based on pore size distribution.

The MIP test method has limitations when it comes to obtaining precise information on pore structure [25]. For example, sample drying is associated with pore structure damage under mercury pressure. Another limitation is the presence of a pore with a large diameter in a sample, wherein the pore is interpreted as the diameter of the small inlet if the inlet to enter is small. Therefore, modification is necessary when pore size distribution as determined using the MIP test is to be used to understand the water absorption phenomenon in mortar samples. To this end, it is assumed that there are small pores which can connect to all the other pores which have a certain diameter. Such a diameter is referred to as the threshold diameter [25]. The threshold diameter is the diameter at which the rate of mercury intrusion according to the diameter is the highest. Hence, the increment at the threshold diameter is regarded as the volume of mercury that can enter the large pores through the small pores with the threshold diameter. The air bubbles in the large pores do not undergo much compression, so water occupies almost no space in the pore, and thus the increment is neglected. In the MIP results, the threshold diameter of Ref. was found to be 674 nm and that of SAP_0.1, SAP_0.2, and SAP_0.3 827–829 nm.

Suppose there is an uncompressed air bubble in a pore. Let *R* be the radius of the air bubble. The radius when the air bubble is compressed by the surface tension of the water is called *R_A_*. The length is expressed in millimeters, the pressure in the uncompressed air bubble is set to 0.1 MPa, the surface tension between the water and the air is set to 7.4 × 10^−5^ N/mm, and the contact angle of the water in the combination of air, water, and mortar is set to zero. The cubic equation for *R_A_* can then be obtained as

*R_A_*^3^ + 1.48 × 10^−3^*R_A_*^2^ − *R*^3^ = 0 (1)

**Figure 9:**
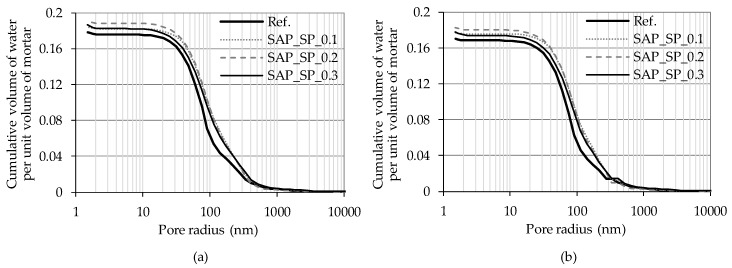
Cumulative volume of water absorbed by a unit volume of mortar. (**a**) Raw MIP data and (**b**) MIP data after modifications were applied in the calculations.

The derivation of Equation (1) can be referred to in [17]. Since the radius of the uncompressed air bubble is determined by the radius of the pore containing the bubble, the volume of water absorbed by a unit volume of the pore along the pore radius can be estimated. The cumulative volume of water absorbed by a unit volume of mortar according to the pore radius is shown in Figure 9. The results based on the raw MIP data are shown in Figure 9a and the results based on the MIP data after modification are shown in Figure 9b. Data presented in Figure 9b well predicts the absorption amount of capillary water considering the limitation of the MIP experiment. Therefore, data in Figure 9b was used for the calculation of water volume in mortar in Table 6.

The influence of the SAP_SPs on water absorption in mortar can be analyzed by comparing the predicted amount of water absorption with the capillary suction test results. In Table 6, the value of (1) is the volume ratio of capillary pores in mortar, which was calculated from the MIP data. The values of (2) and (3) are the total cumulative volume of water absorbed by a unit volume of mortar in Figure 9a,b respectively. The value of (4), which is the amount of water absorption per unit volume of the mortar on the seventh day, was obtained by dividing the measured amount of absorbed water by the bulk density of the mortar in Table 3. For Ref., the value of (4) is larger than the value of (3) by 0.009 because trapped air was dissolved in the water and escaped out of the sample, and the sample further absorbed water. If the amount of water that the sample additionally absorbed as the air escaped the sample is the same for all the samples, the additional water absorption due to the SAP_SPs can be estimated by the formula “value of (4) − value of (3) − 0.009”. The phenomenon in which the dissolved air in water escapes out of the sample is difficult to predict due to the interaction of air with pores of different sizes and the movement of air dissolved in pore water.

Assuming that the SAP_SPs absorbed the increased water uptake, the absorbency of the SAP_SPs can be estimated by dividing “value of (4) − value of (3) − 0.009” by the mass of dry SAP_SPs in a unit volume of mortar in Table 5. The absorbency was estimated to be 23 g/g–44 g/g. This value is much higher than the SAP_SP absorbency of 3.1 g/g in fresh mortar. Because water is almost incompressible, it can be inferred that the water-absorbing SAP_SP will not stay in the space of the air void formed by the SAP_SP but will expand to the relatively large capillary pores or air voids surrounding it. On the other hand, a simple comparison of measured water uptake and capillary porosity shows that Ref. absorbed a smaller volume of water than the capillary porosity but that the samples containing SAPs absorbed a greater volume of water.

### 4.3. Internal Damage

When mortar has absorbed water and the temperature drops below the freezing point, the water in the relatively large capillary pores starts to freeze; as the temperature further decreases, the water in smaller capillary pores freezes [26]. When water transforms to ice, the volume increases by 9%, causing the pressure in the unfrozen water to increase, damaging the mortar [27]. When air remains in the relatively large capillary pores or when air voids are present, water will move there and the water pressure will decrease. Table 2 shows that Ref. had the highest value of air content, at 2.2%. However, this value is also lower than that of the reported samples with excellent freeze–thaw resistance (generally more than 4%). Hence, air voids in Ref. do not effectively reduce water pressure during freezing.

In the case of mixtures incorporating SAP_SP, higher amounts of SAP_SP make the cementitious matrix weaker due to the higher w/c_(effective)_. Thus, the degree of internal damage caused by freeze–thaw cycles was found to increase with higher SAP_SP content in our designed samples.

Furthermore, the SAP_SP in the mortar sample seemed to absorb water and expand into relatively large capillary pores or air voids. This phenomenon causes capillary pores to become filled with water, increasing the amount of water that must be transferred to an air void during freezing. Water-filled air voids do not act as water reservoirs. This phenomenon results in an overall increase in hydraulic pressure. However, the swelling of SAP_SP was lowered in the freeze–thaw resistance test in comparison to the capillary absorption test because NaCl solution was used instead of water.

In the strain measurement test during the freeze–thaw cycles, the mortar samples were exposed to more severe conditions than the samples in the CEN/TS 12390-9 freeze–thaw resistance test due to their small size. Air movement in mortar occurs mainly between differently sized pores, but the shorter the distance between the air and the sample surface, the faster the air escapes from the sample [17]. Hence, the smaller the sample size, the quicker the air escapes and the faster the water is absorbed. In Figure 6, the peak point of the strain during the freezing phase of all samples can be seen to increase as the cycle is repeated. One reason for this is that the mortar samples continuously absorbed water during the cycle. The peak point of the strain of Ref. during the freezing phase of the fourth cycle is larger than that of SAP_SP_0.1, but the strain does not increase with a steep slope. SAP_SP_0.3, which has the highest value of w/c_(effective)_, shows the largest strain, followed by SAP_SP_0.2, during the freezing phase. The strain in both samples can be observed to increase with a steep slope during the freezing phase (of the third cycle in SAP_SP_0.3 and of the fourth cycle in SAP_SP_0.2).

Similar trends are seen in the results for the relative dynamic modulus in the freeze–thaw resistance test. The higher the value of w/c_(effective)_, the faster the relative dynamic modulus decreases as the cycle repeats. This finding indicates that the higher the value of w/c_(effective)_, the more internal damage occurs in the sample as the cycle repeats.

### 4.4. Salt Scaling Damage

When concrete is exposed to both deicing salt and water, freezing and thawing cause damage to the surface of the concrete. The theory of the “glue-spall” model [28] seems to account for the salt scaling phenomenon observed by researchers. Surface damage is in fact related to the deformation of concrete. The thermal expansion coefficients of ice and concrete greatly differ. The coefficient of ice is ~50 × 10^−6^ K^−1^, whereas that of concrete is ~10 × 10^−6^ K^−1^ [28]. The ice layer, which is mechanically attached to the surface of the concrete, is subjected to tensile stress as the temperature decreases. When the tensile stress exceeds a certain level, the ice cracks and the concrete is damaged. When the temperature drops below the freezing point, NaCl solution freezes and forms unfrozen pockets of brine. These pockets make the ice easier to crack. Medium NaCl (2–5 mass %) solution forms a layer of ice with moderate strength to damage the concrete [28].

When concrete shrinks as the temperature decreases below the freezing point, critical tensile stress will not be applied to the ice layer. When the concrete considerably expands, critical tensile stress will be applied to the ice layer and the concrete will also be damaged. Referring to the strain on the mortar sample shown in Figure 6, it can be expected that the higher the SAP_SP content (i.e., the higher the w/c_(effective)_ in our system) the more damage there will be on the concrete surface due to a weaker matrix. This prediction is consistent with the salt scaling results of the freeze–thaw resistance test as shown in Figure 8.

## 5. Conclusions

In this work, a reference mixture and mixtures incorporating SAP with different w/c_(effective)_ and SAP_SP content have been studied with respect to pore size distribution, capillary water absorption, expansion of mixtures under freezing, and freeze–thaw resistance. The fact that the internal damage and salt scaling were found to increase as the SAP_SP content increased can be explained mainly by a weaker cementitious matrix due to the increased w/c_(effective)_ in the samples. In addition, the capillary suction test results showed that the higher the SAP_SP content (i.e., the higher the w/c_(effective)_), the greater the amount of water absorbed in a unit volume of mortar. Assuming that SAP_SP absorbed the increased water uptake, the absorbency of SAP_SP was calculated to be 23 g/g–44 g/g. This value is considerably larger than the SAP_SP absorbency of 3.1 g/g of fresh mortar. Therefore, it can be inferred that water-absorbing SAP_SP does not stay in the air voids formed by SAP_SP but expands to the relatively large surrounding capillary pores or neighbor air voids. In this case, the freeze–thaw resistance may be adversely affected.

## Figures and Tables

**Table 1:** Mix proportions of the samples (mass % of cement).

Sample	Water/Cement Ratio				SAP/Cement Ratio	Raw Materials/Cement Ratio
	w/c_(total)_	w/c_(add)_	w/c_(stored by SAPs)_	w/c_(effective)_		
Ref.	0.45	-	-	0.45	-	Sand (1.6)
SAP_SP_0.1	0.471	0.021	0.003	0.468	0.001	PCE (0.007)
SAP_SP_0.2	0.492	0.042	0.006	0.486	0.002	Gravel (2.5) ^1^
SAP_SP_0.3	0.513	0.063	0.009	0.504	0.003	-

Legend: SAP, superabsorbent polymer; SAP_SP, cross-linked sodium polyacrylate SAP; w/c_(total)_, total water/cement; w/c_(add)_, additional water considering SAP_SP absorbency on the basis of the tea bag test/cement; w/c_(stored by SAPs)_, water stored by SAP considering SAP_SP absorbency on the basis of the flow test/cement; w/c_(effective)_ = w/c_(total)_ − w/c_(stored by SAPs)_; and PCE, polycarboxylate ether. ^1^ Mortar samples did not contain gravel.

**Table 2:** Air content of fresh concrete.

Sample	Ref.	SAP_SP_0.1	SAP_SP_0.2	SAP_SP_0.3
Air Content (%)	2.2	1.9	1.6	1.4

**Table 3:** Bulk density, skeletal density, and porosity of mortar samples as measured by a mercury intrusion porosimetry (MIP) test.

Sample	Ref.	SAP_SP_0.1	SAP_SP_0.2	SAP_SP_0.3
Bulk Density (g/mL)	2.05	2.04	2.01	2.04
Skeletal Density (g/mL)	2.55	2.57	2.54	2.59
Porosity (vol. %)	19.4	20.5	20.8	21.0

**Table 4:** Average peak points of the strains on three specimens for each series during the freezing phase of the fourth cycle.

Sample	Ref.	SAP_SP_0.1	SAP_SP_0.2	SAP_SP_0.3
Strain × 10^6^	193.7	87.4	312.3	466.7

**Table 5:** Mass of dry SAP_SPs and volume of air voids formed by the SAP_SPs in a unit volume of mortar.

Sample	SAP_SP_0.1	SAP_SP_0.2	SAP_SP_0.3
Mass of Dry SAPs in Mortar (mg/mL)	0.36	0.70	1.08
Volume of SAP Air Voids in Mortar (vol. %)	0.11	0.22	0.34

**Table 6:** Calculation procedure for absorbency of SAP_SP during capillary water uptake in mortar.

Sample	Ref.	SAP_SP_0.1	SAP_SP_0.2	SAP_SP_0.3
(1) Capillary Porosity ^1^ (mL/mL)	0.185	0.193	0.198	0.195
(2) Water Volume in Mortar (Figure 9a) (mL/mL)	0.178	0.184	0.191	0.186
(3) Water Volume in Mortar (Figure 9b) (mL/mL)	0.171	0.177	0.183	0.178
(4) Water Volume in Mortar on the Seventh Day (mL/mL)	0.180	0.202	0.209	0.212
(4) − (3) (mL/mL)	0.009	0.025	0.026	0.034
Increased Water Volume Due to SAP_SPs ^2^ (mL/mL)	0	0.016	0.017	0.025
Water Absorbency of SAP_SPs ^3^ (g/g)	-	44	24	23

^1^ Volume ratio of pores with a diameter smaller than 10 μm in a mortar that has capillary pores and air voids. ^2^ Values were calculated using the formula “(4) − (3) − 0.009”. ^3^ Values were calculated by the formula “((4) − (3) − 0.009)/mass of dry SAP_SPs in a unit volume of mortar”.
